# Bacteria Wear ICG Clothes for Rapid Detection of Intracranial Infection in Patients After Neurosurgery and Photothermal Antibacterial Therapy Against *Streptococcus* Mutans

**DOI:** 10.3389/fbioe.2022.932915

**Published:** 2022-07-06

**Authors:** Long Zhang, Deyun Zhang, Hai Tang, Yufu Zhu, Hongmei Liu, Rutong Yu

**Affiliations:** ^1^ Institute of Nervous System Diseases, Xuzhou Medical University, Xuzhou, China; ^2^ Department of Biomedical Engineering, Southern University of Science and Technology, Shenzhen, China; ^3^ Department of Neurosurgery, Affiliated Hospital of Xuzhou Medical University, Xuzhou, China; ^4^ Epilepsy Center, The Affiliated Hospital of Xuzhou Medical University, Xuzhou, China

**Keywords:** bacterial infection, ATP, PTAT, rapid detection, click chemistry

## Abstract

Bacterial infection is one of the most serious physiological conditions threatening human health. There is an increasing demand for more effective bacterial diagnosis and treatment through non-invasive approaches. Among current antibacterial strategies of non-invasive approaches, photothermal antibacterial therapy (PTAT) has pronounced advantages with properties of minor damage to normal tissue and little chance to trigger antimicrobial resistance. Therefore, we developed a fast and simple strategy that integrated the sensitive detection and photothermal therapy of bacteria by measuring adenosine triphosphate (ATP) bioluminescence following targeted photothermal lysis. First, 3-azido-d-alanine (d-AzAla) is selectively integrated into the cell walls of bacteria, photosensitizer dibenzocyclooctyne, and double sulfonic acid-modified indocyanine green (sulfo-DBCO-ICG) are subsequently designed to react with the modified bacteria through *in vivo* click chemistry. Next, the sulfo-DBCO-ICG modified bacteria under irradiation of 808 nm near-infrared laser was immediately detected by ATP bioluminescence following targeted photothermal lysis and even the number of bacteria on the infected tissue can be significantly reduced through PTAT. This method has demonstrated the ability to detect the presence of the bacteria for ATP value in 32 clinical samples. As a result, the ATP value over of 100 confirmed the presence of bacteria in clinical samples for 22 patients undergoing craniotomy and ten otitis media patients. Overall, this study paves a brand new avenue to facile diagnosis and a treatment platform for clinical bacterial infections.

## 1 Introduction

Intracranial infections, especially bacterial infections, are serious and life-threatening complications in patients after neurosurgical procedures and are a major cause of morbidity and mortality (Shofty et al., 2016; Taj and Jamil, 2016; Cao et al., 2018). About 0.8–6.6% of surgical site infection within neurosurgery occurs, accounting for approximately 14% of postoperative deaths (Lepski et al., 2021). Therefore, rapid and accurate intracranial bacterial detection at an early stage is a key to diagnosis and treatment. However, it is hard to get a clear diagnosis of intracranial infection early enough during hospitalization.

Clinical detections of most bacteria are still based on microbial culture. Microbial culture needs a 3–5 days time-consuming process and has an 8–20% low positive rate (Abayasekara et al., 2017; Gajdács et al., 2020). The non-strict application of clinical antibiotics can make the increasing incidence of bacterial multi-resistance (Zhang et al., 2020). Methicillin-resistant *Staphylococcus aureus* (*MRSA*) has given rise to several difficult-to-treat infections in recent years (Grinberg et al., 2017; Pei et al., 2018; Wang et al., 2019). Cerebrospinal fluid (CSF) biochemical and routine tests usually yield rapid results. However, due to the existence of intraoperative aseptic inflammatory reactions on various indicators, the diagnostic value has not yet reached a consensus. Distinguishing between intracranial infections and non-infectious is still challenging. Therefore, it is urgent to develop a timely and accurate detection method and effective therapy for bacterial infection.

Photothermal antibacterial therapy (PTAT), utilizing a photosensitizer to convert the absorbed light energy into heat for the eradication of pathogens, has been widely developed (Wu et al., 2019). Owing to its high efficiency, non-invasiveness, and low systemic toxicity, PTAT has become one of the most desirable strategies to combat bacterial infections (Shi et al., 2020). More importantly, PTAT exhibits significant antibacterial effectiveness in drug-resistant bacteria compared with traditional antibiotic therapy (Cao et al., 2020). Indocyanine green (ICG) is a near-infrared (NIR) dye for clinics approved by the U.S. Food and Drug Administration (FDA) (Tan et al., 2017; Thawani et al., 2017; Yim et al., 2018; Jia et al., 2019). It is the safest for the human body due to ICG can bind to plasma proteins and exclude them from blood in 20 min after intravenous injection. ICG not only can be used for NIR fluorescence but also can convert the absorbed light energy to local hyperthermia for PTT (Li et al., 2015; Li et al., 2016; Wan et al., 2018; Li W. et al., 2019). Many studies used ICG fluorescence and photothermal properties for tumor images and therapies (Guerrero et al., 2017; Zhang et al., 2017; Wu et al., 2018; Zhang et al., 2019a). Meanwhile, some researchers used ATP bioluminescence following photothermal lysis to detect the bacteria (Vanithamani et al., 2015; Li X. et al., 2019). The measurement of ATP, a molecule found in living cells, *via* bioluminescent firefly luciferin/luciferase reaction has been widely employed for the detection of pathogenic bacteria. This method is based on luciferase-based detection of ATP, which results in the production and emission of bioluminescent light *via* enzymatic reaction. The bioluminescent signal is directly proportional to the amount of ATP released from lysed bacteria (Turner et al., 2010). Whether using the properties of ICG may achieve the detection and treatment of bacterial infection.

d-amino acids are the main component of the bacterial wall and can be metabolically expressed on the bacterial wall. Previous studies have reported that azide-modified d-alanine (3-azido-d-alanine, d-AzAla) can selectively target bacteria and express on the bacterial wall to expose the azide functional group (Yang et al., 2018; Miyamoto et al., 2019). Azide group can react with dibenzocyclooctyne modified molecular by biorthogonal reaction (Mao et al., 2018). Thus, we wonder if we used the bacterial metabolic biomolecular labeling method to give bacteria wearing clothes for diagnosing and treatment of bacterial infection.

**FIGURE 1:**
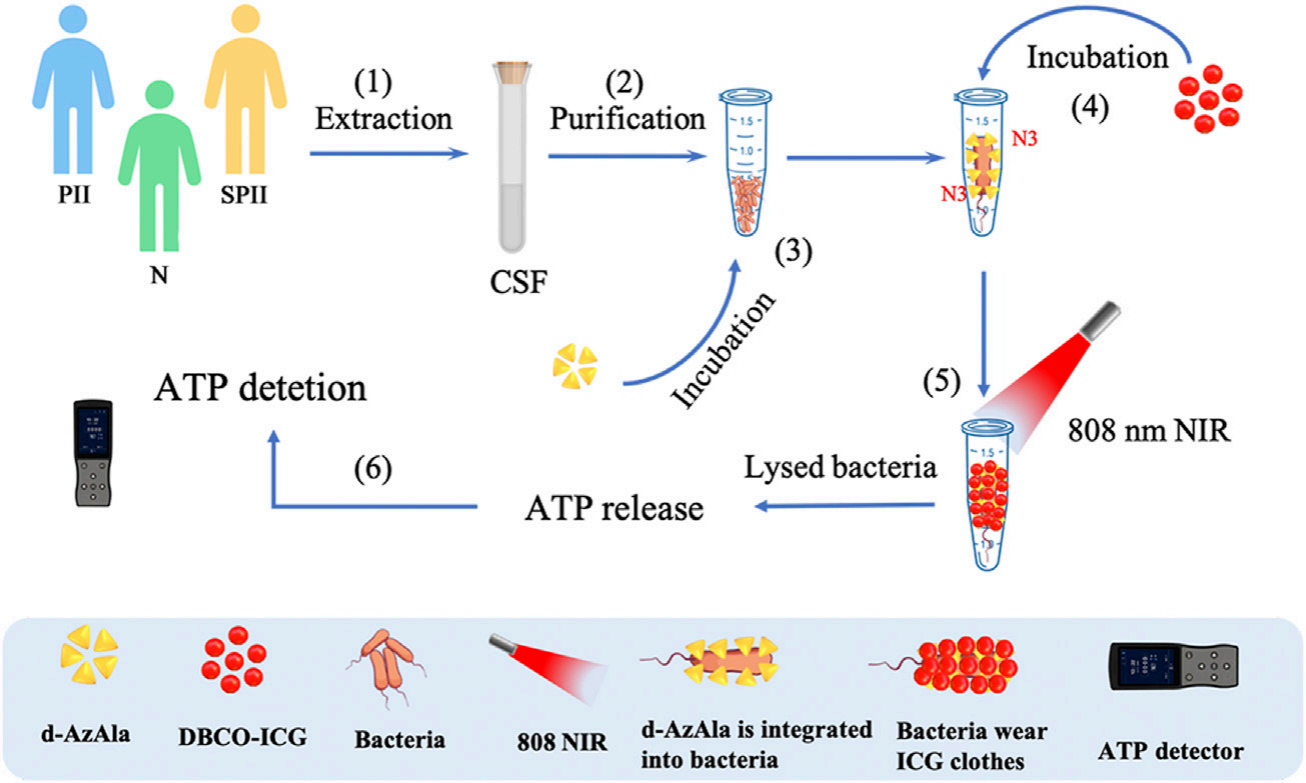
Detection of bacteria using sulfo-DBCO-ICG reacted with d-AzAla modified bacteria by copper-free click chemistry-mediated photothermal lysis and measurement of ATP bioluminescence. (1) Extraction of CSF from patients (postoperative with intracranial infection (PII), suspected postoperative with intracranial infection (SPII), normal (N)). (2) Purification of CSF to remove red blood cells. (3) Feeding of d-AzAla to bacteria and the cell walls of bacteria to carry N3 groups. (4) The sulfo-DBCO-ICG was then incubated with N_3_-modified bacteria, and the sulfo-DBCO-ICG reacted with the modified bacteria through click chemistry, formed the bacteria wear ICG clothes. (5) An 808 nm laser was used to illuminate bacteria wear ICG clothes. (6) ATP-based bioluminescence methods were utilized for the bacteria detection.

In this study, we developed a method to let bacteria wear ICG clothes to realize timely ATP, fluoresces double detections, and PTAT for bacterial infection (Figure 1). Soluble DBCO-ICG reacted with d-AzAla modified bacteria by copper-free click chemistry to achieve ICG-coated bacteria (ICG-bacteria). Bacteria wore ICG clothes, which had three advantages: 1) ICG fluorescence was tested to detect the bacteria. 2) Under an 808 nm laser, the bacteria were lysed to release ATP at a high temperature. Then, ATP-based bioluminescence methods were utilized for the rapid detection of intracranial infection. 3) Sulfo-DBCO-ICG could effectively accumulate in the bacterial infection area to coat bacteria. After irradiation by the 808 nm laser, ICG-induced photothermal antibacterial therapies effectively eradicated bacteria and provided an accelerated healing process. This method does not cause any drug resistance as caused by antibiotics and is not toxic/harmful to adjacent normal tissues.

## 2 Materials and Methods

### 2.1 Materials

3-Azido-d-alanine hydrochloride (d-AzAla) was purchased from baseclick GmbH, Inc. Sulfo-DBCO-ICG was purchased from Biosyntech, Inc. (Suzhou, China). ATP-eliminating reagents were purchased from BioThema, Inc. *Escherichia coli* (*E. coli*, ATCC 25922) and *MRSA* (ATCC 43300) were obtained from Yasong Biotechnology, Ltd. (Nanjing, China). Indocyanine green was obtained from Tokyo Chemical Industry Co., Ltd (Tokyo, Japan). An ATP fluorescence detector was obtained from Xi’an Tianlong Science and Technology Co., Ltd.

### 2.2 Methods

#### 2.2.1 Bacteria Culture

*E. coli* (ATCC 25922) and *MRSA* (ATCC 43300) were incubated in tryptic soy broth (TSB) medium in a shaking incubator (200 rpm) at 37°C overnight. For each experiment, a single colony of *E. coli* and *MRSA* was selected from the fresh TSB agar plate and subjected to the culture at 37°C and 200 rpm overnight. Finally, the cultured bacteria were introduced for further experiments.

#### 2.2.2 Confocal Laser Scanning Microscope and Flow Cytometry of Sulfo-DBCO-ICG-bacteria

Bacterial cell suspensions were diluted to obtain cell samples containing 1 × 10^6^ to 1 × 10^7^ colony-forming units (CFU) mL^−1^. In 1.5 ml microcentrifuge tubes, we mixed 250 μl of bacterium suspensions with 40 μl of 1 mg ml^−1^ DBCO-ICG and 100 μl of d-AzAla. Then, the mixture (DBCO-ICG-bacteria) was incubated in an incubator in the dark at 37°C for 1 h. Ten microliters of the stained bacterial suspension were trapped on an 18 mm square coverslip. We observed them in confocal laser scanning microscopy (CLSM, LSM880, Germany). In the same method, DBCO-ICG-bacteria were fixed in 4% paraformaldehyde. All samples were run on LSRFortessa, and the data were analyzed using FlowJo v9.9.8. The excitation/emission of the dyes was 780–810 nm for the ICG.

#### 2.2.3 Morphological Characterization of Bacteria

For the morphological characterization of bacteria, the bacterial suspensions of the abovementioned treatment groups were fixed overnight with a 2.5% glutaraldehyde solution after the assessment of antibacterial properties. Then they were dehydrated by sequential treatments with 30, 50, 70, 80, 90, and 100% of ethanol successively for 10 min. After drying with a critical point dryer, all samples were sputter-coated with platinum for SEM observation. Scanning electron microscopic images were recorded using a Tecnai G2 T12 instrument from FEI Company (United States). TEM images were recorded using an FEI TECNAI G220 high-resolution transmission electron microscope operating at 200 kV.

#### 2.2.4 Antimicrobial Activity Measurement

A 10^5^ CFU ml^−1^ bacteria suspension was incubated with different samples at room temperature for 15 min in the dark. Then, we centrifuged the sample, discarded the supernatant, centrifuged it again, and irradiated it with 808 nm laser (1.0 W cm^−2^, LDF-II, Beijing Laserwave Optoelectronics Technology Company, China) for 90 s. The obtained bacterial solutions were spread on sterile agar plates and incubated upside at 37°C. The number of CFUs was counted after 24 h, and the photographs of the formed bacterial colonies were taken. Bacterial solution without any treatment in the dark was used as a control. Bacterial viability was calculated using the following equation:

Bacterial viability=C test/C control×100%.

Here, C test and C control correspond to the CFU number for the test group and control group, respectively.

#### 2.2.5 ATP Bioluminescence-Based Bacterial Detection Following Photothermal Lysis

Bacteria (200 μl) prepared were mixed with d-AzAla and DBCO-ICG successively and then incubated at room temperature for 30 min. Thereafter, solutions were irradiated for 90 s using an 808 nm laser (1.0 W cm^−2^). The resulting solution was added to a luciferin−luciferase solution, and bioluminescence was immediately measured using a microplate reader (Xian Tianlong Technology Company, China). The bioluminescent signal was measured in less than 30 s.

#### 2.2.6 Ethical Approval of CSF of Clinical Patients

For the collection of CSF of clinical patients, ethical approval was obtained from the Ethics Committee of the Affiliated Hospital of Xuzhou Medical University (Ethics identifier XYFY2020-KL209-01, Chairperson Prof Tie Xu), Jiangsu, China.

#### 2.2.7 Inclusion Criteria of Clinical Patients

For patients undergoing craniotomy with clinically confirmed intracranial infections, their results of multiple bacterial cultures were positive. Patients with suspected intracranial infection and with negative CSF culture results showed symptoms of infection, which were persistent high fever and the routine cell count in CSF >1 × 10^7^ L^−1^. Patients without intracranial infection (such as Parkinson’s disease and communicating hydrocephalus) were negative in CSF culture and had a white blood cell count of less than 10 × 10^9^ L^−1^.

#### 2.2.8 Detection of ATP Value in CSF of Patients With Craniotomy

First, we collected CSF samples from the patients, and then we used cell lysing reagent and ATP eliminating reagent to remove ATP outside the bacteria. Next, we added azide alanine and added DBCO-ICG. Finally, we discarded the supernatant by centrifugation.

#### 2.2.9 Animal Care and Maintenance

6–8 weeks ICR male mice (30–40 g) were obtained from Beijing HFK Bioscience Co., Ltd. (Beijing, China). All animals were placed in an SPF-grade IVC barrier system and bred adaptively for 7 days. All animal experiments were approved by the Xuzhou Medical University of China Animal Care and Use Committee.

#### 2.2.10 Establishment of *MRSA*-Infected Mouse Models

Animal experiment procedures were carried out in keeping with the Guidelines for Care and Use of Laboratory Animals of Xuzhou Medical University. On day 4 before the infection, the mice were administered one dose of CTX. 150 mg CTX per kg mouse body weight (150 mg kg^−1^) was injected i. p. This treatment fostered a more vulnerable environment in the mice to infection. The mice were anesthetized using a standard anesthesia procedure, and then the dorsal region of the mice was shaved to prepare for surgery. Then one round wound of 80 mm in diameter was made using a puncher on the left back of mice weighing 30–40 g with 5 ICR male mice in each group. After 6 h, 50 μl of *MRSA* (1 × 10^8^ CFU ml^−1^) was slowly added to each wound.

#### 2.2.11 *In Vivo* Antimicrobial Assay

After the establishment of *MRSA*-infected mouse models, the infection sites were treated with drugs and the same volume of PBS *via* the tail vein. PTAT treatment was conducted by irradiating the infection sites with laser irradiation (808 nm, 1.0 W cm^−2^) for 180 s. The infection sites were treated with PTAT solutions every 2 days. The regeneration process of wounds was studied through wound area monitoring and histomorphological determination. In wound size measurement, the mice in each group were anesthetized, and the wound size was determined by tracing the boundaries of wounds on days 3, 7, 11, and 14. For histomorphological evaluation, wound tissue of day 14 was collected for biochemical analysis. The samples were made into 0.5 cm^2^ square shape and immersed in standard formalin solution. Then tissue samples were conducted with H&E staining and prepared into a wax section for observation.

#### 2.2.12 Statistical Analyses

All data were collected in triplicate, and statistical analysis was performed with SPSS version 23.0. Statistical comparisons of data from the experiments were performed using Student’s t-test with two tails or one-way ANOVA for multiple comparisons, followed by Dunnett’s *t*-test for *post hoc* pairwise comparisons. *p-*values <0.05 were considered statistically significant. The experimental results are given in the format of mean ± SD in the figures (∗∗∗*p* < 0.001, ∗∗*p* < 0.01, and ∗*p* < 0.05).

## 3 Results

### 3.1 Characterization of Sulfo-DBCO-ICG Binding to D-AzAla-Bacteria

**FIGURE 2:**
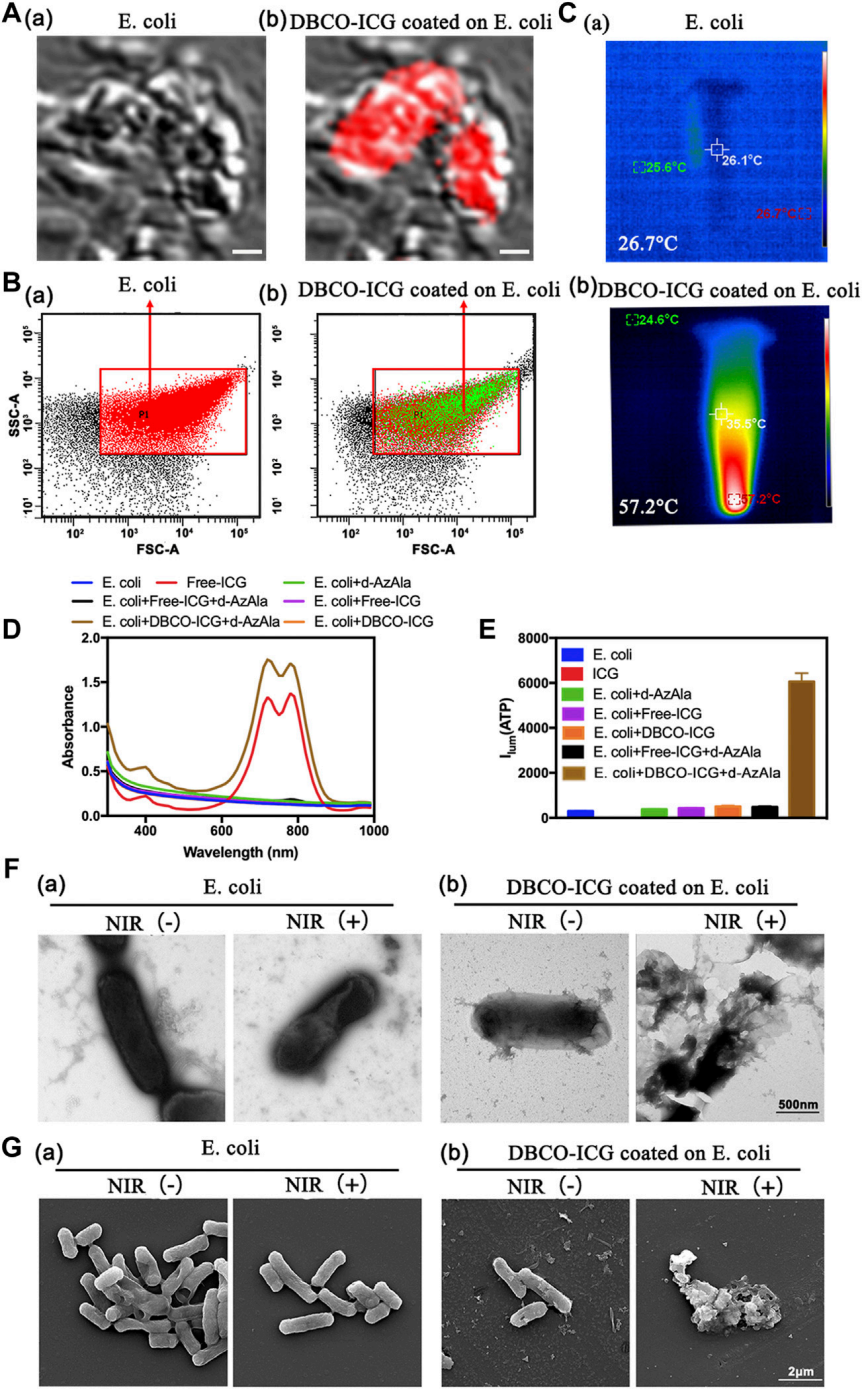
Sulfo-DBCO-ICG effectively coated on d-AzAla-bacteria and measured the ATP bioluminescence from the lysed bacteria. **(A)** CLSM images of *E. coli* (a) and D-ICG coated on *E. coli* (b). **(B)** Flow cytometric quantification of conjugation with *E. coli* by DBCO-ICG **(C)** Photothermal images of *E. coli* (a) and DBCO-ICG coated on *E. coli* after 90s (b). **(D)** UV–Vis-NIR absorbance spectra of *E. coli*, Free-ICG, *E. coli* + d-AzAla, *E. coli* + Free-ICG, *E. coli* + DBCO-ICG, *E. coli* + d-AzAla + Free-ICG, and *E. coli* + d-AzAla + DBCO-ICG. **(E)** ATP detector measure the ATP bioluminescence of *E. coli*, Free-ICG, *E. coli* + d-AzAla, *E. coli* + Free-ICG, *E. coli* + DBCO-ICG, *E. coli* + d-AzAla + Free-ICG, and *E. coli* + d-AzAla + DBCO-ICG. **(F)** TEM images of bacteria following NIR irradiation. (a) Bacteria in the absence of DBCO-ICG conjugates. (b) Targeted photothermal lysis of bacteria by DBCO-ICG conjugates in the presence and absence of NIR irradiation. **(G)** SEM images of bacteria following NIR irradiation. (a) Bacteria in the absence of DBCO-ICG conjugate. (b) Targeted photothermal lysis of bacteria by DBCO-ICG conjugates in the presence and absence of NIR irradiation. The laser used in all the aforementioned measurements is an 808 nm NIR laser with a power intensity of 2 W cm^−2^.

Sulfo-DBCO-ICG was functionalized by conjugated with d-AzAla-bacteria. Therefore, we tested if sulfo-DBCO-ICG was effectively coated on d-AzAla-bacteria by confocal laser scanning microscope (CLSM), flow cytometry, and other experiments. As shown in Figure 2A, sulfo-DBCO-ICG-bacteria had ICG fluorescence detected by CLSM. The same result was confirmed by a flow cytometry assay. Strong ICG fluorescence signals of sulfo-DBCO-ICG-bacteria were tested with the flow cytometer, suggesting that ICG successfully reacted with d-AzAla-bacteria in contrast to only bacteria (without ICG fluorescence) (Figure 2B). As shown in Figure 2C, the UV-vis absorption spectra of sulfo-DBCO-ICG effectively coated on d-AzAla-bacteria exhibited a slightly red-shift compared with pristine ICG from 779 to 795 nm mainly attributed to the interactions between sulfo-DBCO-ICG and d-AzAla-bacteria. ICG can produce heat upon 808 nm NIR irradiation, which can make bacteria photothermal lysis. The photothermal performance of only bacteria and sulfo-DBCO-ICG-bacteria was investigated under an 808 nm laser. The maximum temperature of ICG-bacteria reached 57.2°C for 90 s irradiations, whereas the temperature of only bacteria was 26.7°C (Figure 2D). Next, we briefly verified whether sulfo-DBCO-ICG heat production could cause the effective release of ATP after bacterial lysis by an ATP detector. From Figure 2E, after NIR irradiation, the sulfo-DBCO-ICG coated on the d-AzAla-bacteria group released a large amount of ATP, while the other groups released almost low ATP due to ICG producing thermal-induced bacteria cracking under the 808 nm NIR irradiation. The morphological changes of ICG-bacteria under the 808 nm infrared laser irradiation were observed by transmission electron microscopy (TEM) and scanning electron microscopy (SEM). As shown in Figures 2F and G, the morphology of sulfo-DBCO-ICG-bacteria was changed and lysed after irradiated 808 nm NIR Figures 2F and G (b). As shown in Figures 2F and G (a) bacteria were not bound to DBCO-ICG, and the morphology of the bacteria did not change with or without NIR irradiation. It also indirectly proved that the morphological change of bacteria is very slight or even below 808 nm infrared laser irradiation. All these abovementioned results suggest that sulfo-DBCO-ICG effectively coated on d-AzAla-bacteria and could produce heat to crack bacteria for releasing ATP under 808 nm NIR irradiation.

### 3.2 *In vitro* Rapid Detection of Bacteria

**FIGURE 3:**
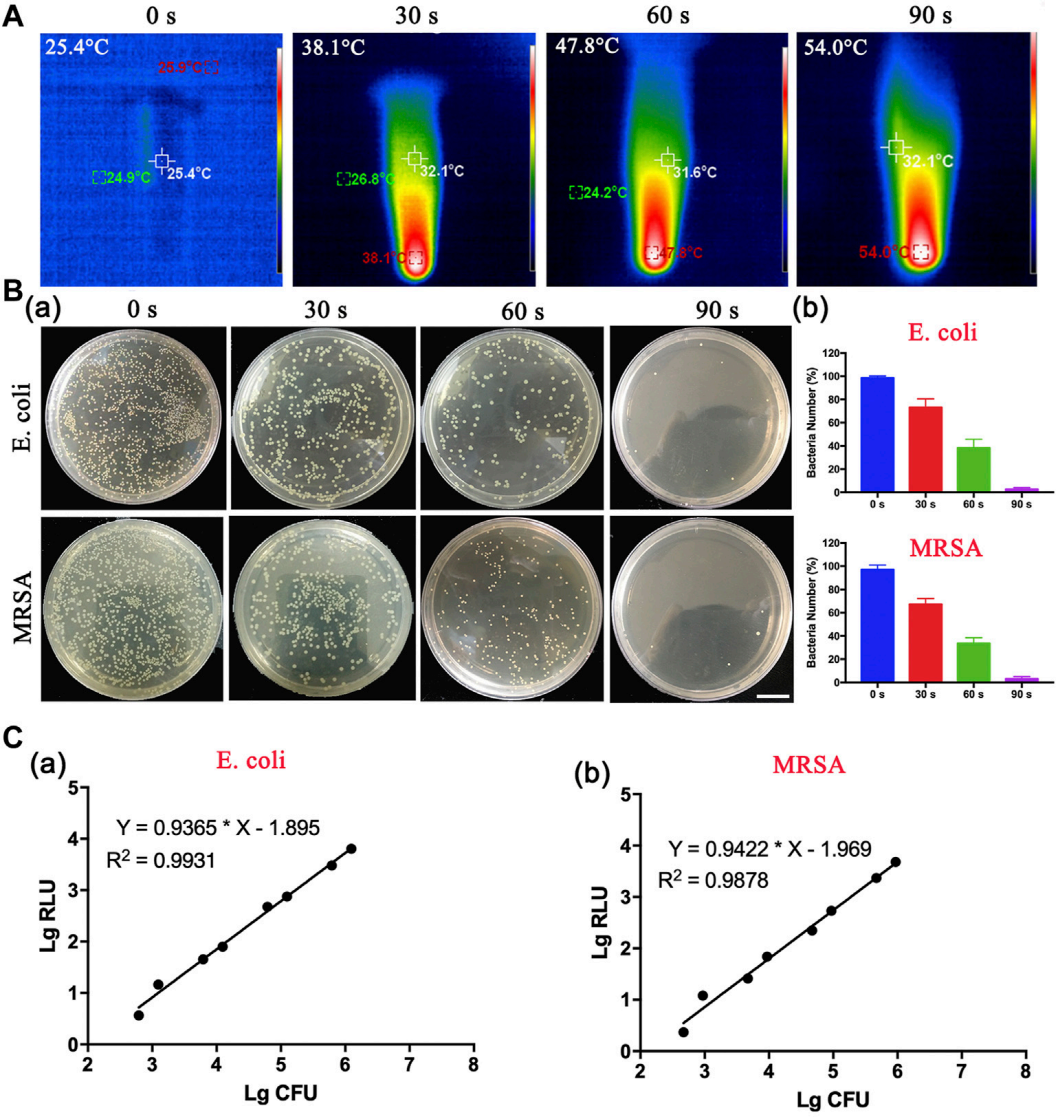
*In vitro* thermogenesis and antibacterial function of sulfo-DBCO-ICG. **(A)** Detection of photothermal properties of DBCO-ICG irradiated by 808 nm laser. **(B)** The pyrolysis of bacteria under different irradiation times. (a) The colony growth of *E. coli* and *MRSA* on the after 808 nm NIR. (b) The survival rate of bacteria after *E. coli* and *MRSA* were irradiated by 808 nm NIR. **(C)** Correlation between RLU and CFU of *E. coli* (a) and *MRSA* (b) *in vitro*.

Motivated by these results, we speculated that DBCO-ICG was a detection reagent for the rapid detection of bacteria. The photothermal properties of the DBCO-ICG coated on bacteria were examined under 808 nm NIR by a thermal imager. As illustrated in Figure 3A, the heat production gradually increased with prolonging irradiation duration with the DBCO-ICG-*E. coli* heat production temperature reached 54°C after 90 s of irradiation. In order to observe the effect of bacterial lysis after heating, a bacterial spread plate assay was conducted. The bacterial colony counts steadily decreased when the irradiation time of 808 nm NIR was increased from 0 to 30, 60, and 90 s (Figure 3Ba). In particular, when irradiation time of 808 nm NIR reached 90 s, there were almost no colonies of bacteria on the Luria–Bertani (LB) agarose solid medium, suggesting that all bacteria died by thermal cracking. The bacterial survival rate decreased significantly after 60 s of irradiation, which was 39.8% and 34.6%, respectively. When the irradiation time reached 90 s, the bacterial survival rate was only 0.94% (*E.coli*) and 0.98% (*MRSA*), showing a good bacterial thermal cracking effect (Figure 3B). For subsequent detection of bacteria, ATP value was tested after NIR irradiation for 90 s. The quantification of the correlation between bacterial colony count and ATP value was tested. As shown in Figure 3C, there was a certain linear relationship between the colony-forming unit (CFU) of bacteria and the relative light unit (RLU) of ATP value. The results showed a positive correlation between the log-adjusted CFU and RLU for both *E. coli* (Figure 3Ca) and *MRSA* (Figure 3Cb), and this correlation was statistically significant. ATP value had a dynamic range with a multiple-log scale of linearity (Supplementary Table S1). These results demonstrate that this method of measuring bacteria ATP for evaluation of intracranial infection in patients after neurosurgery is feasible.

### 3.3 Rapid Detection of Intracranial Infection in Patients After Neurosurgery

**FIGURE 4:**
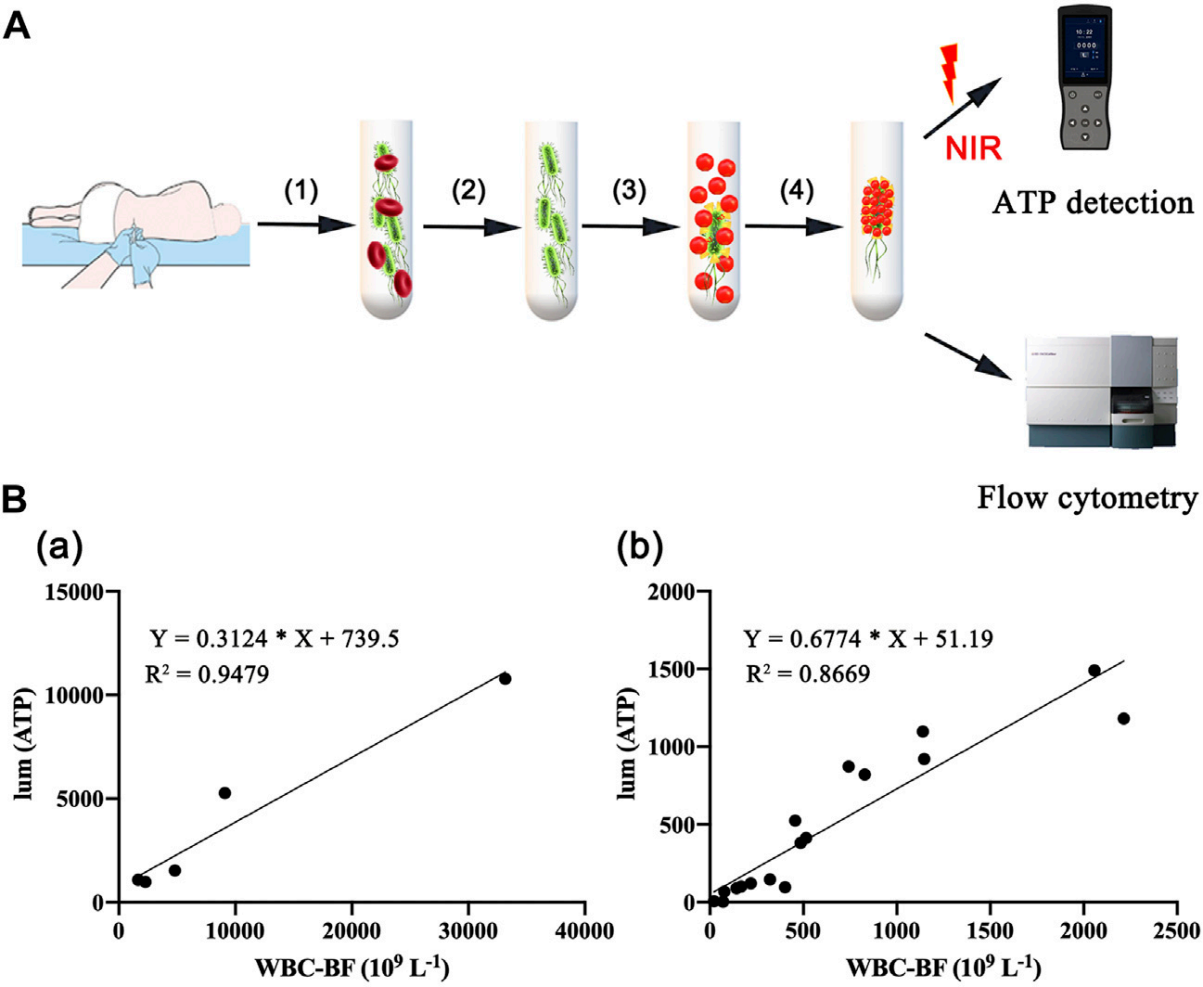
Rapid detection of intracranial infection in patients. **(A)** Detection of ATP in CSF samples from 30 patients, including five patients with clinically confirmed intracranial infections, 17 patients with suspected intracranial infection, and eight patients without intracranial infection. **(B)** Correlation analysis between white blood cell count and ATP value in CSF. (a) CSF of five patients with confirmed postoperative intracranial infection. (b) CSF of 17 patients with suspected postoperative intracranial infection.

Most bacteria are detected by microbial culture in the clinic; however, the long duration of the microbiological culture process and the low positive rate make it difficult for patients to diagnose intracranial infection early during hospitalization (Overmann et al., 2017; Hohnadel et al., 2018). Based on the experimental results of our *in vitro*, it was verified that the DBCO-ICG efficiently coated on d-AzAla-bacteria and could produce heat to fracture bacteria for the releasing ATP below 808 nm NIR irradiation. In order to quickly diagnose intracranial infection in patients after neurosurgery, we collected CSF from patients undergoing craniotomy for ATP testing (Figure 4A). Of the 22 patients who met the inclusion criteria, five patients were clinically confirmed with intracranial infection by a cultured bacterium after surgery (PII), and 17 patients were suspected with intracranial infection after surgery (SPII, with fever, suspected with bacteria). We also collected CSF from eight Parkinson’s disease and communicative hydrocephalus patients who were not infected (normal, without open craniotomy). Five patients with confirmed intracranial bacterial infections had high ATP values (more than values of 900) (Table 1), while eight patients without infection had low ATP values (less than values of 12 and even values of 0) (Table 2), suggesting that DBCO-ICG effectively coated on bacteria in CSF samples and quickly detected them. Then, with the exception of two patients, we utilized the same method to identify the CSF of 17 patients with suspected intracranial bacterial infection, and the ATP value was greater than 60 (Table 3). Based on the aforementioned ATP value of bacteria detection *in vitro*, 15 patients in suspected bacterial infection groups had high ATP values, demonstrating that 15 patients had brain infection and the bacteria could not be cultivated properly. Some researchers reported that white blood cell count can predict bacterial infection; a high white blood cell count is usually a symptom of infection (Anandalwar et al., 2015; Riley and Rupert 2015; Li et al., 2020; Tascini et al., 2020). The linear correlation between ATP value and the number of white blood cells in the blood was evaluated. As shown in Figure 3B, the linear correlation coefficient of 5 patients with bacterial infection was *R* ^2^ = 0.9479, indicating that there was a strong correlation between ATP value and white blood cells (Figure 4Ba). The linear correlation coefficient of 17 patients with suspected intracranial bacterial infection was *R* ^2^ = 0.8669, demonstrating that there was a strong correlation between ATP value and white blood cells (Figure 4Bb). From these results, we speculated that these 17 patients were infected by bacteria. Altogether, our method realized accurate and rapid detection of bacteria after neurosurgery.

**TABLE 1:** Data on CSF samples from five patients with confirmed postoperative intracranial infection.

Number	WBC-BF (× 10^9^ L^−1^)	Glucose (mmol L^−1^)	HsCRP (mg L^−1^)	Protein (g L^−1^)	Positive/Negative	ATP Value
1	2,383.00	3.35	15.7	1.30	Positive	980
2	1,664.00	—	—	—	Positive	1,078
3	4,807.00	1.63	46.4	17.49	Positive	1,526
4	9,110.00	2.43	13.4	2.90	Positive	5,274
5	33,164.00	1.11	30.6	4.50	Positive	10,783

**TABLE 2:** Data on CSF samples from eight patients without intracranial infection.

Number	WBC-BF (× 10^9^ L^−1^)	Glucose (mmol L^−1^)	HsCRP (mg L^−1^)	Protein (g L^−1^)	Positive/Negative	ATP Value
1	2.00	3.19	—	0.43	Negative	2
2	2.00	3.58	—	0.20	Negative	2
3	2.00	2.86	—	0.30	Negative	3
4	1.00	4.04	—	0.43	Negative	8
5	2.00	5.10	—	0.60	Negative	12
6	0.00	2.83	—	0.20	Negative	0
7	8.00	3.08	—	0.40	Negative	2
8	1.00	4.96	—	0.60	Negative	4

**TABLE 3:** Data on CSF samples from 17 patients with suspected postoperative intracranial infection.

Number	WBC-BF (× 10^9^ L^−1^)	Glucose (mmol L^−1^)	HsCRP (mg L^−1^)	Protein (g L^−1^)	Positive/Negative	ATP Value
1	21.00	2.13	14.1	4.38	Negative	5
2	70.00	3.37	22.3	1.01	Negative	3
3	77.00	5.47	37.2	18.96	Negative	68
4	142.00	3.33	10.1	1.80	Negative	89
5	167.00	4.01	29.1	3.69	Negative	101
6	219.00	3.99	22.3	1.40	Negative	121
7	322.00	5.82	8.70	1.20	Negative	146
8	402.00	4.50	21.1	4.80	Negative	96
9	456.00	4.29	49.9	2.30	Negative	524
10	486.00	3.63	15.3	1.08	Negative	381
11	514.00	1.74	8.9	6.10	Negative	412
12	742.00	4.87	6.6	1.10	Negative	872
13	829.00	4.17	71.4	1.70	Negative	820
14	1,139.00	1.11	14.8	3.40	Negative	1,097
15	1,147.00	3.41	13.2	1.05	Negative	920
16	2058.00	2.75	13.4	3.50	Negative	1,490
17	2,214.00	4.76	16.6	4.21	Negative	1,180

Our approach is not only useful for the rapid detection of bacterial infection after neurosurgery but also for detecting infected bacteria in other situations. Middle ear effusions (MEE) were collected from ten otitis media patients and were detected bacterial infection by our method. Supplementary Table S2 shows that five of the ten patients with otitis media had a high ATP value, suggesting that they were infected with bacteria. The above-stated results were in line with the hospital’s identification of microbiological cultures. ICG is a widely used clinical fluorescent dye that can be excited by absorption light in the wavelength range of 750–810 nm. Therefore, flow cytometry might detect the fluorescence of DBCO-ICG-bacteria to identify whether or not there is a bacterial infection. As shown in Supplementary Figure S1, CSF from patients without bacterial infection showed little fluorescence (Supplementary Figure S1A), whereas CSF from infected patients had obvious fluorescence changes under DBCO-ICG incubation (Supplementary Figure S1B), indicating that this fluorescence detection method may achieve rapid detection of infected bacteria. Overall, our method could detect bacteria rapidly through ATP and fluorescence detection.

### 3.4 Evaluation of Targeting Ability *in vivo*

**FIGURE 5:**
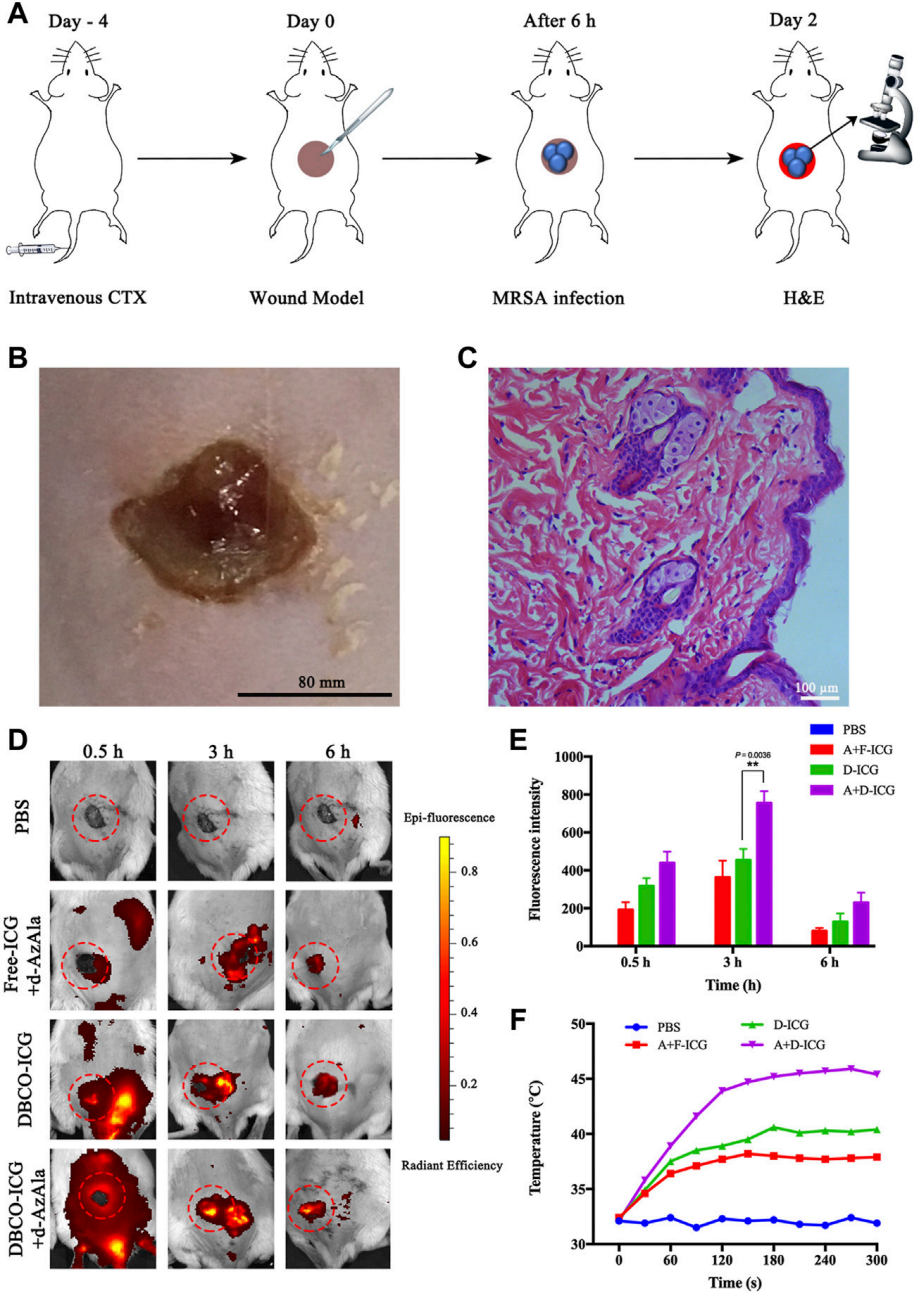
Appraised the targeting ability of DBCO-ICG *in vivo*. **(A)** Schematic diagram of the establishment of the *MRSA*-infected model. **(B)** Skin for *MRSA*-infected model. **(C)** *MRSA*-infected skin for H&E staining. **(D)** *In vivo* imaging of ICG to assay the targeting ability of DBCO-ICG. **(E)** Bioluminescence signal quantification in *MRSA*-infected model (n = 5) (*p < 0.05). **(F)** Temperature time curve of PBS, Free-ICG + d-AzAla, DBCO-ICG, and DBCO-ICG + d-AzAla in the *MRSA*-infected skin region after 808 nm laser irradiation.

Motivated by the aforementioned results, we concluded that DBCO-ICG was a type of prospective antibacterial agent that may be used in photothermal therapy for bacterial infections. The model was *MRSA*-infected mice with wounds on their backs, which were used in the *in vivo* imaging assays. On day 4 before the establishment of the *MRSA*-infected model, cyclophosphamide (CTX) was injected intravenously at a concentration of 150 mg ml^−1^ (Wang et al., 2018). The mouse’s own immune system was suppressed by CTX medicines, allowing the mouse to better infect its own environment. (Ahlmann and Hempel 2016). The cut injury of mice and the subcutaneous abscesses induced by local vaccination of *MRSA* were conducted as shown in Figure 5A. After 2 days of local *MRSA* inoculation on the back of the mice, we observed obvious redness and swelling of the skin on the back from Figure 5B. Meanwhile, we stained the *MRSA*-infected epidermis with hematoxylin-eosin (H&E) (Figure 5C) and measured the amount of WBC in blood from the *MRSA*-infected model’s tail vein (Supplementary Figure S2). The results showed that the *MRSA*-infected skin was infiltrated by a large number of inflammatory cells and the number of WBC in the *MRSA* group was significantly higher than that of normal mice. These results could clearly prove that our infection model is successfully established.

Then, evaluation of targeting ability was used in the *in vivo* imaging assays. In the verification of the target experiment, four groups were used to treat *MRSA*-infected mouse wounds: PBS, Free-ICG + d-AzAla, DBCO-ICG, and DBCO-ICG + d-AzAla. They were photographed in animal imagers at 0.5, 3, and 6 h after intravenous administration of several ICGs to observe the targeting impact of each treatment. As shown in Figures 5D and E, the DBCO-ICG + d-AzAla group markedly increased the distribution of encapsulated fluorophore in *MRSA*-infected skin compared with Free-ICG + d-AzAla group at each time point. In particular, the strongest fluorescence intensity could be observed in the DBCO-ICG + d-AzAla group at 3 h point (**p = 0.0036). We also discovered that Free-ICG in the body is not only untargeted but has a quick metabolism, which is consistent with the literature (Lim et al., 2014). On the contrary, DBCO-ICG fluorescence still appeared around the infected wound after 6 h, indicating that we added a DBCO group to Free-ICG, which not only gives ICG a click chemical reaction with d-AzAla but also prolongs ICG metabolism in the body. Next, we irradiated the infected wound of the *MRSA*-infected model with near-infrared after 3 h of administration to see if it could efficiently produce heat. As shown in Figure 5F, it was observed that after 180 s of near-infrared light in the DBCO-ICG + d-AzAla group, the temperature of the infected skin on the back of the mouse could reach 46°C, and bacteria could be effectively lysed at this temperature (Yang et al., 2019; Shen et al., 2020), but the temperature of other groups only increased to about 38°C. The above findings show that DBCO-ICG could effectively target the infection wound in the *MRSA*-infected model and could attain temperatures high enough to kill bacteria when exposed to 808 nm NIR irradiation.

### 3.5 *In Vivo* Antibacterial and Biocompatibility

**FIGURE 6:**
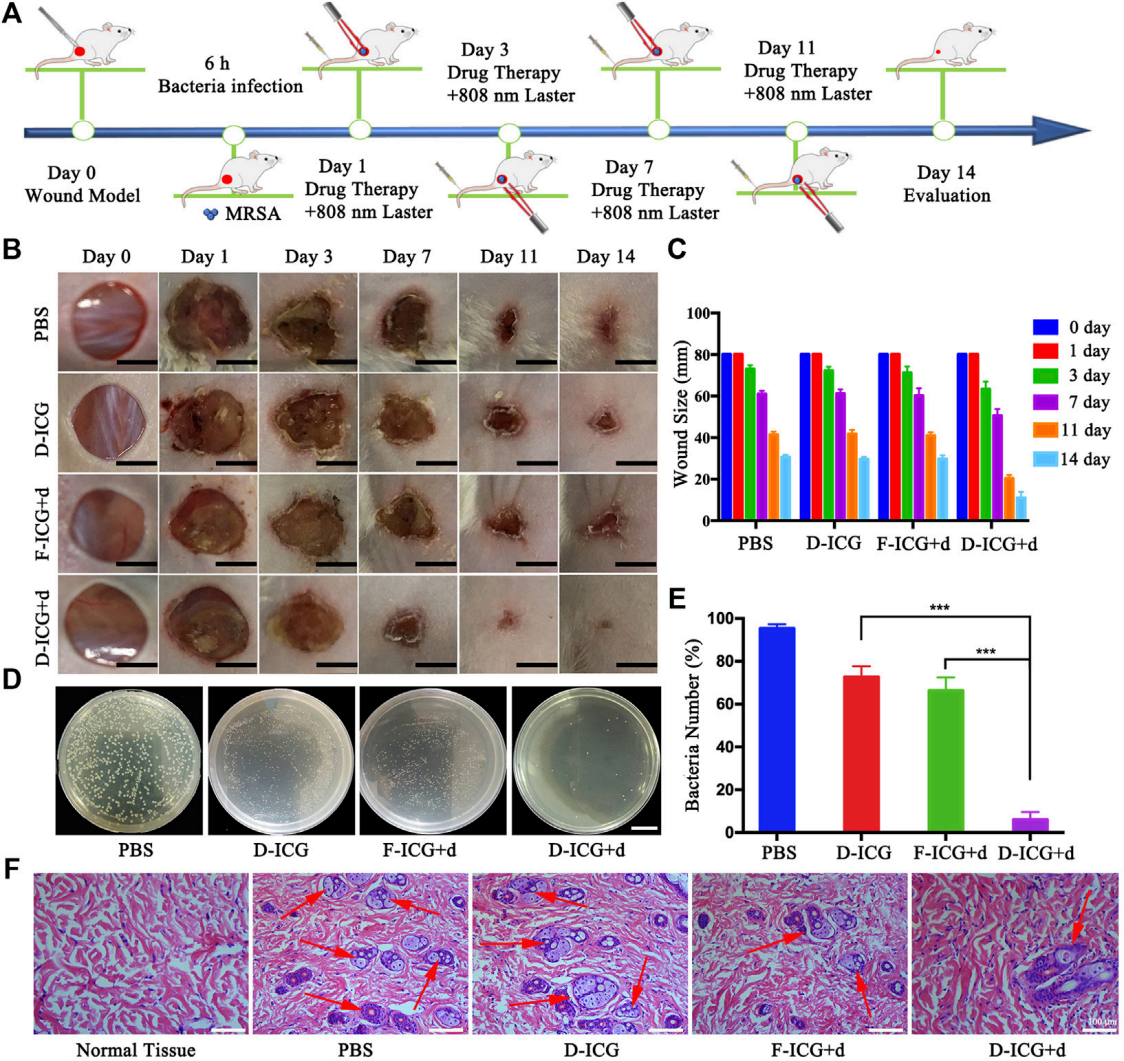
*In vivo* antibacterial effect on treating subcutaneous abscess of *MRSA*-infected mice. **(A)** Schematic diagram of the cut injury and the following *MRSA* vaccination. **(B)** Changes in wounds size from day 1 to day 14, each group of mice was severally treated with PBS, D-ICG, F-ICG + d, or D-ICG + d; scale bars are 50 mm. **(C)** Wound size (%) on different days (1, 3, 7, 11, and 14) (n = 5). **(D)** Corresponding statistical results of colony counts in wound tissue (***p < 0.001, **p < 0.01, *p < 0.05). **(E)** Number of bacteria on the wound skin tissues using the agar plate dilution. **(F)** Photomicrographs showing sections of wound tissues with H&E staining. Inflammatory cells are marked by red arrows. Scale bars are 100 μm.

After confirming drug targeting and thermogenesis *in vivo*, we began to evaluate the antibacterial efficacy of DBCO-ICG in *MRSA*-infected mice wounds. Dorsal wounds induced on the back of mice were topically treated with PBS + NIR, DBCO-ICG (D-ICG) + NIR, Free-ICG + d-AzAla (F-ICG + d) + NIR, and DBCO-ICG + d-AzAla (D-ICG + d) + NIR, respectively. The cut injury of mice and the subsequent *MRSA* incubation were conducted as shown in Figure 6A. We evaluated the antibacterial therapeutic effect of the drugs in each group based on the wound healing and the number of bacteria on the back skin wounds after 2 weeks. As shown in Figures 6B and C, we could see that the back wounds of all kinds of mice healed after treatment, but the degree of healing was not consistent. The D-ICG + d treated group noticeably had less scar, which was much better than other groups, suggesting that the D-ICG + d treated group could distinctly accelerate wound healing and had the best bactericidal effect. Then, we also could see that there was no difference between the treatment effects of groups F-ICG + d and D-ICG, and the effect was not as good as the D-ICG + d group. This phenomenon suggests that Free-ICG was not targeted *in vivo*, could not form an azide reaction with amino acids on the surface of bacteria, and also reflects the poor antibacterial effect of DBCO-ICG alone. It must allow bacteria to wear ICG clothes and fluorescence double detections and PTAT for bacterial infection. DBCO-ICG reacted with d-AzAla modified bacteria by copper-free click chemistry to achieve ICG-coated bacteria. Meanwhile, on day 14, we conducted another investigation utilizing the agar plate dilution method to detect the number of bacteria on the wound skin tissues. There is no doubt that D-ICG + d treated group had exceptional antibacterial activity; the D-ICG + d group was able to destroy around 95% of *MRSA* (***p < 0.001) (Figures 6D and E). In addition, the wound that had been treated differently were stained with H&E and photographed. Figure 6F shows images of the four groups stained with H&E, and large inflammatory cells (neutrophils, red arrow) appeared in the others group. However, inflammatory cells were scarce in the D-ICG + d treated group. This result was consistent with our observations from H&E of normal skin tissue, which shows that the D-ICG + d group has excellent antibacterial properties and can promote wound healing. Finally, the systemic biosafety of the DBCO-ICG was assessed *in vivo*. Compared with the PBS group, the H&E staining of the main organs treated with Free-ICG + d-AzAla, DBCO-ICG, and DBCO-ICG + d-AzAla had no noticeable tissue damage and changes in morphology, indicating that the DBCO-ICG above had no obvious biological toxicity (Supplementary Figure S3). These results demonstrate that DBCO-ICG showed an effective antibacterial effect in the *MRSA*-infected model, and no appreciable toxic side effects observed were induced by the DBCO-ICG treatments.

## 4 Discussion

Bacterial infections are the most common type of clinical infection. Bacterial infectious diseases are increasingly threatening the health of global people due to the abuse of clinical antibiotics (Mostafalu et al., 2017; Dong et al., 2020). In particular, surgical site infection within neurosurgery occurs, accounting for approximately 14% of postoperative deaths (Shi et al., 2017). Clinical detections of most bacteria are still based on microbial culture. However, the long duration of the microbiological culture process and the low positive rate make it difficult for patients to diagnose intracranial infection early during hospitalization (Zhang et al., 2019b). In the first essential diagnostics list (EDL) released by the World Health Organization in 2018, it was highlighted that rapid, sensitive, specific, and reasonably priced diagnostics have a fundamental role in tailoring appropriate treatments of infectious diseases caused by bacteria (Tang et al., 2019). Therefore, our study developed a fast and simple strategy for the sensitive detection of bacteria by measuring ATP bioluminescence following targeted photothermal lysis.

ICG, a near-infrared dye, is approved by the U.S. Food and Drug Administration as a clinical diagnosis agent (Su et al., 2015). ICG has been extensively explored for PTAT and NIR fluorescence imaging due to its NIR optical characteristics within the ideal absorption window for biomedical applications (Li et al., 2014). According to a previous research, d-amino acids are a component of peptidoglycan, the principal component of the bacterial cell wall that surrounds bacteria (Typas et al., 2012; Liechti et al., 2014). Bacteria could incorporate it into their cell wall while mammalian cells could not, and azide group can react with dibenzocyclooctyne modified molecular by the biorthogonal reaction. Thus, we utilized d-amino acid as molecules to selectively target bacteria. As shown in Figures 2A and B, sulfo-DBCO-ICG effectively coated d-AzAla-bacteria. DBCO-ICG coated bacteria were lysed and effectively released ATP under 808 nm NIR for 90 s. The correlation between bacterial colony count and ATP values was then quantified. As shown in Figure 3, there is a positive correlation between bacterial colony count and ATP values (the relative coefficient between >0.8).

As a conventional manipulation after neurosurgery, CSF samples were extracted as a useful routine for biochemical detection and cultured to hunt for bacteria from patients with fever of unclear origin after neurosurgery. In addition, WBC, C-reactive protein (CRP) concentrations, and glucose in the CSF are the traditional indicators for preliminary detection of infections and noninfectious conditions. However, the long duration of the clinical microbiological culture process and insensitivity of WBC to infection results in a slow diagnosis of intracranial infection after surgery. Based on our experimental results *in vitro*, it is verified that the sulfo-DBCO-ICG effectively coated on d-AzAla-bacteria and the strong linear relationship between ATP values and CFU (Supplementary Table S1). CSF samples were collected from five patients undergoing craniotomy with clinically confirmed intracranial infections and 17 patients with suspected intracranial infection. ATP detection of CSF was performed using the same method as *in vitro*. Combined with clinical biochemical indicators and the ATP value, the results revealed that the ATP value was non-infected in the range of 0–60, and more than 60 tended to be infected (Table 1 and Table 3). The same results were found in otitis media patients (Supplementary Figure S1).

Finally, the redness and swelling of the skin and the *MRSA*-infected skin for H&E staining confirmed that the *MRSA*-infection model was successfully established (Figure 5B and Figure 5C). The *in vivo* imaging assays proved the targeting ability of the DBCO-ICG in the *MRSA*-infected model, and the temperature of the infected skin on the back of the mouse could reach 46°C after 180 s of near-infrared light of the DBCO-ICG group (Figure 5F). In addition, the *in vivo* imaging assays also verified the good antibacterial effect and biocompatibility of DBCO-ICG in *MRSA*-infected mouse wounds (Figure 6B and Supplementary Figure S2).

## 5 Conclusion

In summary, our study developed a simultaneous detection and antibacterial platform for bacterial infection. It is a fast and simple strategy for the sensitive detection of bacteria by measuring ATP bioluminescence following targeted photothermal lysis. Sulfo-DBCO-ICG reacted with d-AzAla modified bacteria by copper-free click chemistry to complete a precise and rapid target. The sulfo-DBCO-ICG effectively coated on d-AzAla-bacteria and could produce heat to crack bacteria for releasing ATP below 808 nm NIR irradiation *in vitro*. The sulfo-DBCO-ICG could rapidly detect intracranial infections in patients after neurosurgery through ATP detection and flow cytometry. The sulfo-DBCO-ICG has targeting ability, good antibacterial effect, and biocompatibility in the *MRSA*-infected model. This method is faster than the clinical microbiological culture process, does not cause any drug-resistance as caused by antibiotics, and is not toxic/harmful to healthy cells. It may provide a good idea for the clinical rapid detection of bacteria.

## Data Availability

The original contributions presented in the study are included in the article/Supplementary Material; further inquiries can be directed to the corresponding authors.
