# Supplementary material for: Bacteria Wear ICG Clothes for Rapid Detection of Intracranial Infection in Patients After Neurosurgery and Photothermal Antibacterial Therapy Against Streptococcus Mutans
Source: Front Bioeng Biotechnol. 2022 Jul 6;10:932915. doi: 10.3389/fbioe.2022.932915 (PMC9298881; doi:10.3389/fbioe.2022.932915)
Supplement: Supplementary file 1 [file DataSheet1.pdf]

Supplementary Material

Bacteria wear ICG clothes for rapid detection of intracranial infection in patients after neurosurgery and photothermal antibacterial therapy against Streptococcus mutans

Long Zhang[1#], Deyun Zhang[1#], Hai Tang[3], Yufu Zhu[1*], Rutong Yu[1*], Hongmei Liu[2*]

[1] Institute of Nervous System Diseases, Xuzhou Medical University, Xuzhou 221002, P. R. China

[2] Department of Biomedical Engineering, Southern University of Science and Technology, Shenzhen, Guangdong 518055, P. R. China

[3] Epilepsy Center, The Affiliated Hospital of Xuzhou Medical University, Xuzhou 221002, P. R. China

*** Correspondence:**

* Corresponding author: Hongmei Liu, Rutong Yu and Yufu Zhu.

E-mail addresses: liuhm@sustech.edu.cn (Hongmei Liu), yu.rutong@163.com (Rutong Yu), fugle99@163.com (Yufu zhu).

[#] These authors contributed equally to this work.

1.

1 Supplementary Figures and Tables

1.1 Supplementary Figures

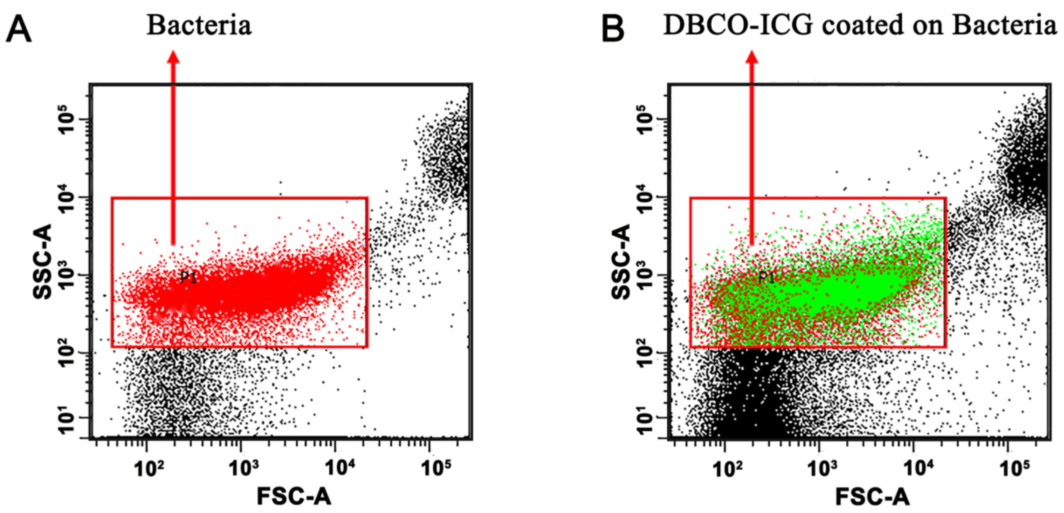

Figure S1. Flow cytometric quantification of conjugation with CSF of patients with intracranial infections after neurosurgery by DBCO-ICG. A. Bacteria of flow cytometric quantification. B. DBCO-ICG coated on bacteria of flow cytometric quantification.

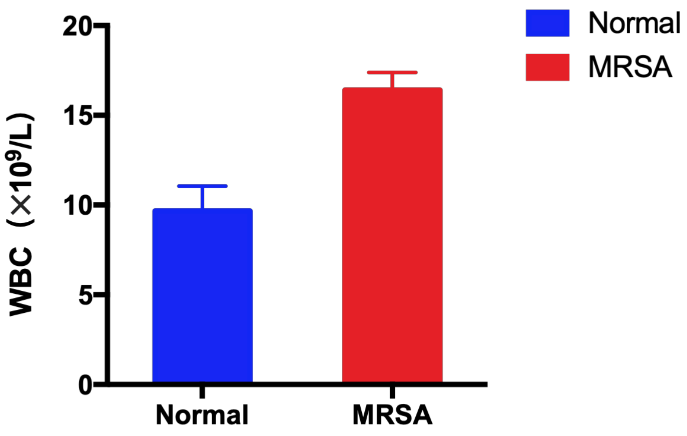

Figure S2. The number of WBC mice in the normal and MRSA-infected groups.

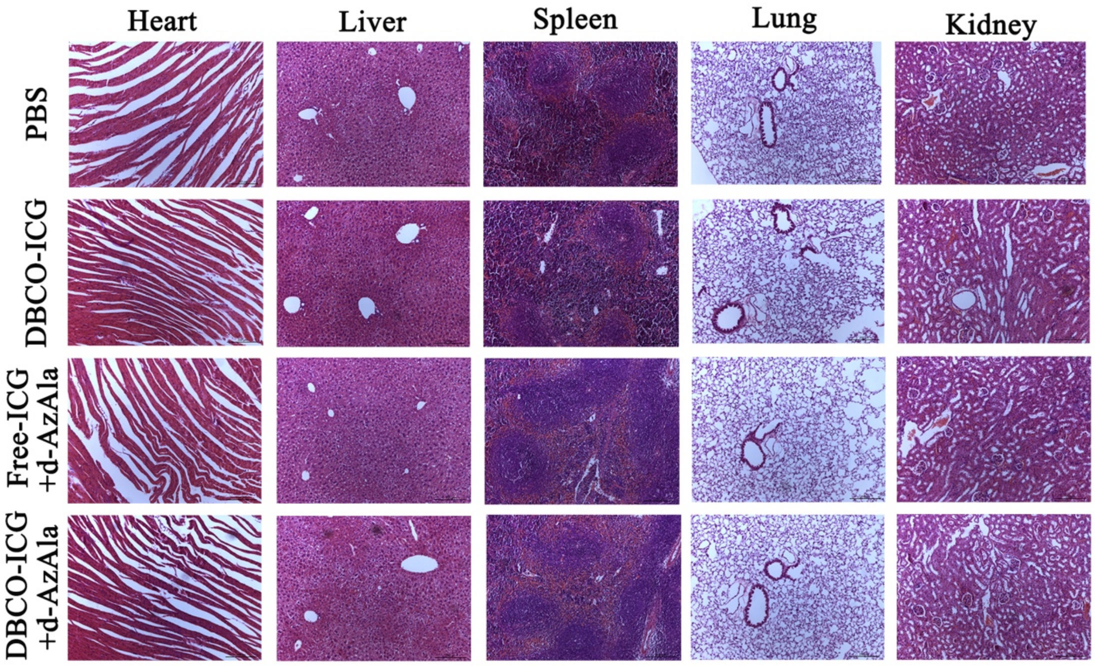

Figure S3. H&E staining of Heart, Liver, Spleen, Lungs and Kidneys treated with PBS, DBCO-ICG, Free-ICG + d-AzAla and DBCO-ICG + d-AzAla. Scale bar = 200 μm.

1.2 Supplementary Tables

Table S1. Correlation between ATP value and dilution of E. coli and MRSA

Dilution ratio of bacteria	ATP value of E. coli	ATP value of MRSA
0.5×10^{-4}	2	4
1.0×10^{-4}	4	9
0.5×10^{-3}	15	27
1.0×10^{-3}	25	55
0.5×10^{-2}	113	367
1.0×10^{-2}	235	889
0.5×10^{-1}	1253	3112
1.0×10^{-1}	2689	6877

Table S2. Date on secretion samples from 10 patients with Otolaryngological Disease

Number	Result of microbial culture	ATP Value
**1**	No bacteria growth	6
**2**	No bacteria growth	10
**3**	No bacteria growth	4
**4**	No bacteria growth	1
**5**	No bacteria growth	15
**6**	Bacteria detected (Streptococcus)	3550
**7**	Bacteria detected (Staphylococcus epidermidis)	4685
